# The influence of maternal factors on the neonatal microbiome and health

**DOI:** 10.21203/rs.3.rs-2485214/v1

**Published:** 2023-02-02

**Authors:** Bin Zhu, Myrna Serrano, Gregory Buck

**Affiliations:** Virginia Commonwealth University; Virginia Commonwealth University; Virginia Commonwealth University

**Keywords:** Neonatal microbiome, maternal microbiome, oral microbiome, gut microbiome, vaginal microbiome, rectal microbiome, NICU, microbial infection, lipidomic, cytokine

## Abstract

The human microbiome plays an essential role in human health. However, the influence of maternal factors on the neonatal microbiome remains obscure. Herein, our observations suggest that the neonatal buccal microbiome is similar to the maternal buccal microbiome, but the neonatal gastrointestinal microbiome develops a unique composition at an early stage. The low complexity of the neonatal buccal microbiome is a hallmark of maternal and neonatal health, but that of the neonatal gastrointestinal microbiome is associated with maternal inflammation-related metabolites. Microbial infections in the maternal reproductive tract universally impact the complexity of the neonatal microbiomes, and the body site is most important in modulating the composition of the neonatal microbiomes. Additionally, maternal lipids attenuated the adverse influence of several maternal factors on the neonatal microbiomes. Finally, admission of neonates to the newborn intensive care unit is associated with sub-optimal states of the maternal buccal and rectal microbiomes and maternal health.

## Introduction

At an early stage of life, gut and oral microbiomes seem to influence immune, metabolic, and other human developmental pathways^1–3^. A sub-optimal early-life gut microbiome is associated with multiple adverse outcomes, including but not limited to obesity^4^, diarrheal disease^5^, Crohn’s disease^6^, type 1 diabetes^7^, and necrotizing enterocolitis^8^. Thus, it is important to investigate the mechanisms of establishing early-life microbiomes.

Immediately after birth (≤ 5 minutes), a vertical mother-neonate microbe transmission has been reported for vaginal deliveries^9,10^; i.e., the neonatal skin, oral and nasal microbiomes are similar in composition to the maternal vaginal microbiome^11^. In contrast, the microbiomes of Cesarean section (C-section) neonates are more likely to resemble the maternal skin microbiome^11,12^. C-section impacts the neonatal nasal, oral, and skin microbiomes but not the meconium microbiome at an early-life stage^3,11, 13–17^, but this influence is lost by six (6) weeks after birth^13^. In both vaginal and C-section deliveries, the abundance of the commonly transmitted taxa seems to decrease with age^9,18^. These results illustrate that at least a part of the maternal microbiome seems only transiently to seed neonatal microbiomes, and the impact of the maternal microbe seeding on the neonatal microbiomes diminishes after birth. By six weeks after birth, the composition and function of the infant’s skin, oral, nasal, and stool microbiomes have been expanded and diversified^13^.

Within three days postpartum, the number of strict anaerobic taxa in the oral and stool microbiomes decreases rapidly^9^, implying that environmental conditions in the neonate have more oxygen exposure than the maternal sites contribute to the microbiome. In contrast, the relative abundance of facultative anaerobes in the stool microbiome increases^9^, presumably mediating the transition from aerobic to anaerobic conditions in the gut^19–21^. The neonatal stool microbiome increases in richness immediately after birth^11^ and continues to increase throughout the first three years of life^22^, indicating a continuous input of microbes from the environment.

Mode of delivery^3^, breast or formula feeding^14^·^23^, time in the newborn intensive care unit (NICU)^24^, and other environmental factors have also been shown to impact the neonatal microbiomes. However, the mechanisms by which maternal factors impact the neonatal microbiomes remain obscure. A previous study^25^ showed that oral administration of maternal vaginal microbes at birth failed to restore the stool microbiome in neonates born by C-section, implying that other factors than seeding from the maternal microbiome modulate the neonatal stool microbiome.

Herein, we examined the maternal and neonatal microbiomes, the demographic and clinical metadata, and the maternal lipidomic and cytokine profiles of 164 mother-neonate dyads previously enrolled in the Multi Omic Microbiome Study Pregnancy Initiative^26,27^. The maturation of the neonatal oral, rectal, and stool microbiomes during the first three days postpartum was characterized, and models of how these changes were directed were proposed. Finally, maternal factors associated with the NICU admission of neonates were investigated.

## Results

### Relationships among the maternal and neonatal microbiomes

The studied dataset was collected from 164 mother-neonate dyads, including 16S rRNA taxonomic profiles from neonatal buccal (NB), rectal (NR), and stool (NS) sites, maternal buccal (MB), rectal (MR), and vaginal (MV) niches in the pregnant women, maternal and neonatal clinical and other metadata, and lipid and cytokine expression levels in the vaginal fluid of the pregnant women. The experimental designs and case numbers are shown in Fig. S1 and Supplementary Data 1 and 2.

Complexity (alpha diversity) quantified by the Shannon index, evenness of bacterial abundance distribution, and the number of observed taxa of the NB and NR microbiomes were lower than those of the MB and MR microbiomes, respectively (Fig. S2), illustrating that the neonatal microbiomes are less complex than the mature maternal microbiomes^22^.

**Figure 1:**
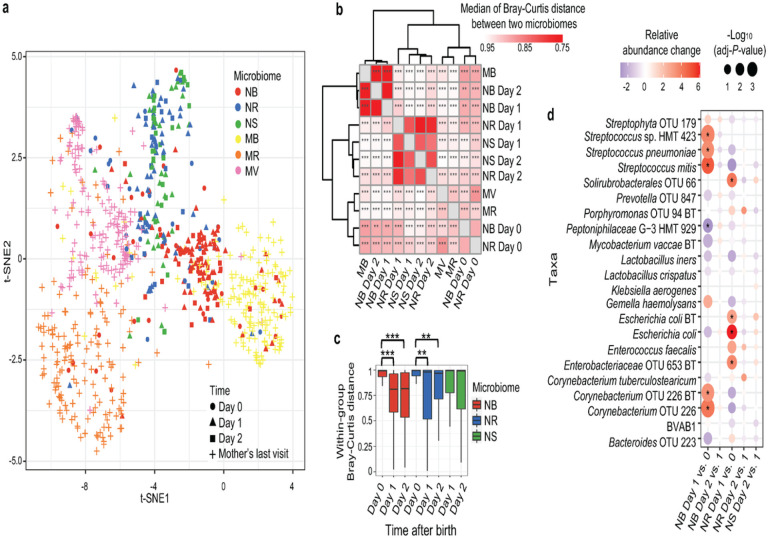
Beta diversity of the neonatal microbiomes within three days postpartum. The neonatal buccal (NB), rectal (NR), and stool (NS) microbiomes on day 0 (within 24 hours postpartum), day 1 (24~48 hours postpartum), and day 2 (48~72 hours postpartum) after birth and the maternal buccal (MB), rectal (MR), and vaginal (MV) microbiomes collected on the last visit of pregnancy were used in the beta diversity analyses. The experimental design is shown in Fig. S1a and Supplementary Data 1 sheet 2. **(a)** Beta diversity of the NB, NR, and NS and MB, MR, and MV quantified by the Bray-Curtis distance and visualized by the t-distributed stochastic neighbor embedding plot. (**b)** A heatmap shows the dissimilarity of the microbiomes evaluated and clustered according to the median values of the Bray-Curtis distances between two microbiomes. The difference between each microbiome dyad was measured by the Adonis test, and the significance is indicated by asterisks. ** *P*-value ≤ 0.01, and *** *P*-value ≤ 0.001. (**c)** Comparison of the within-group Bray-Curtis distance of the neonatal microbiomes using the multiple response permutation procedure test. ** *P*-value ≤ 0.01, and *** *P*-value ≤ 0.001. (**d)** The significant changes in the relative abundance of taxa in the neonatal microbiomes are shown by a dot plot and are highlighted by asterisks. The relative abundance changes of taxa that are abundant in any studied microbiome are also visualized. Relative abundance change was quantified by the per-feature median difference between two conditions. Adjusted *P*-values were generated by the Benjamini-Hochberg correction of the Mann-Whitney U test.

Dissimilarity (beta diversity) of the neonatal and maternal microbiomes was visualized in a t-distributed stochastic neighbor embedding (t-SNE) plot (Fig. 1a and and Fig. S1a) and quantified in a heatmap showing median values of Bray-Curtis distance between each of the paired microbiomes (Fig. 1b). A significant difference in beta diversity between the NB and NR microbiomes was not observed on day 0, but appeared on days 1 and 2 (Fig. 1b and Fig. S3a). In contrast, the within-group dissimilarity of both the NB and NR microbiomes decreased on days 1 and 2 (Fig. 1d). These results are consistent with a previous report^13^ suggesting that the NB and NR microbiomes rapidly diversify from each other but that the microbiomes at each body site tend to converge among individuals after birth (Supplementary Movie 1).

Compared to the maternal microbiomes, the neonatal microbiomes generally clustered more closely on the t-SNE plot (Fig. 1a), suggesting a higher similarity among the neonatal microbiomes consistent with a shared source of taxa at the beginning of life. Recent reports have suggested that the NB microbiome is similar to the MV microbiome immediately after birth (≤ 5 minutes)^11^. Herein, on day 0 (≤ 24 hours), the NB microbiomes were more widely distributed on the t-SNE plot, and some of the NB microbiomes clustered among each of the three maternal microbiomes (Fig. 1a). These observations imply that microbes in the NB microbiome within 24 hours may derive from different maternal sources. The NB microbiome on day 0 was most similar to that of the MB microbiome (Fig. 1b). Consistent with previous observations^23^, the differences between the NB and MB microbiomes decreased with time, but the same phenomenon was not observed between the NR and MR microbiomes (Fig. S3b-d, and Supplementary Movie 1). These observations suggest that the composition of the NB microbiome tends to converge on the MB microbiome after childbirth, but the NR microbiome develops a composition that diverges from the MR microbiome at an early stage. However, the NB-MB distance in the paired mother-neonate dyads was not higher than in unpaired samples (Fig. S3e), indicating that general maternal factors shared by all the women were not primary factors modulating the composition of the NB microbiomes. The beta diversity of the NR microbiome on day 0 was most similar to that of the MV microbiome (Fig. 1b), suggesting that the main source of the initial NR microbiome may be the MV microbiome.

### Composition of the neonatal microbiomes

Consistent with beta diversity analysis, the predominant taxa in the NB and MB microbiomes were the same Gram-positive and aerobic or facultative anaerobic bacterial taxa, e.g., *Corynebacterium* OTU 226, *Streptococcus mitis,* and *Streptophyta* OTU 179 (Fig. S4 and Supplementary Data 3). The predominant taxa in the NR microbiome on day 0 were similar to those abundant in the MV and MB microbiomes. However, the composition of the NR microbiome on days 1–2 differed from any of these three maternal microbiomes but was more similar to the NS microbiome; i.e., the dominant taxa were *Escherichia coli* and other *Enterobacteriaceae* spp.

Differential abundance analysis using ‘ALDEx2’^28^ showed that the relative abundance of the predominant taxa on days 1 and 2 and several closely related taxa, e.g., several *Corynebacterium* and *Streptococcus* spp. in the NB and *Enterobacteriaceae* spp. in the NR, were increased from day 0 to day 1 (Fig. 1d and Supplementary Data 4). However, no significant changes were observed from day 1 to day 2. These results suggested that the composition of the NB and NR microbiomes changed abruptly within 24–48 hours but were more stable by the third day postpartum. Furthermore, network analysis showed that predominant taxa, e.g., *Streptococcus* and *Corynebacterium* spp. in the NB microbiome (Fig. S5) and *Enterobacteriaceae* spp. in the NR microbiome (Fig. S6), had negative correlations with taxa abundant in other body sites in related microbiomes. Since the predominant taxa in the NB and NR microbiomes had increased relative abundance from day 0 to day 1 (Fig. 1d), it was not surprising that many of the taxa abundant at other body sites had reduced relative abundances from day 0 to day 1, although these abundance changes were not significant (Fig. 1d and Supplementary Data 4).

### Matemal And Neonatal Factors Associated With The Neonatal Microbiome Structure

Maternal metadata, microbiomes, lipids, and cytokines on the last visit of pregnancy and paired neonatal metadata and microbiomes on the first visit after birth were selected to identify factors that are associated with the neonatal microbiomes (Fig. S1b and Supplementary Data 1).

**Figure 2:**
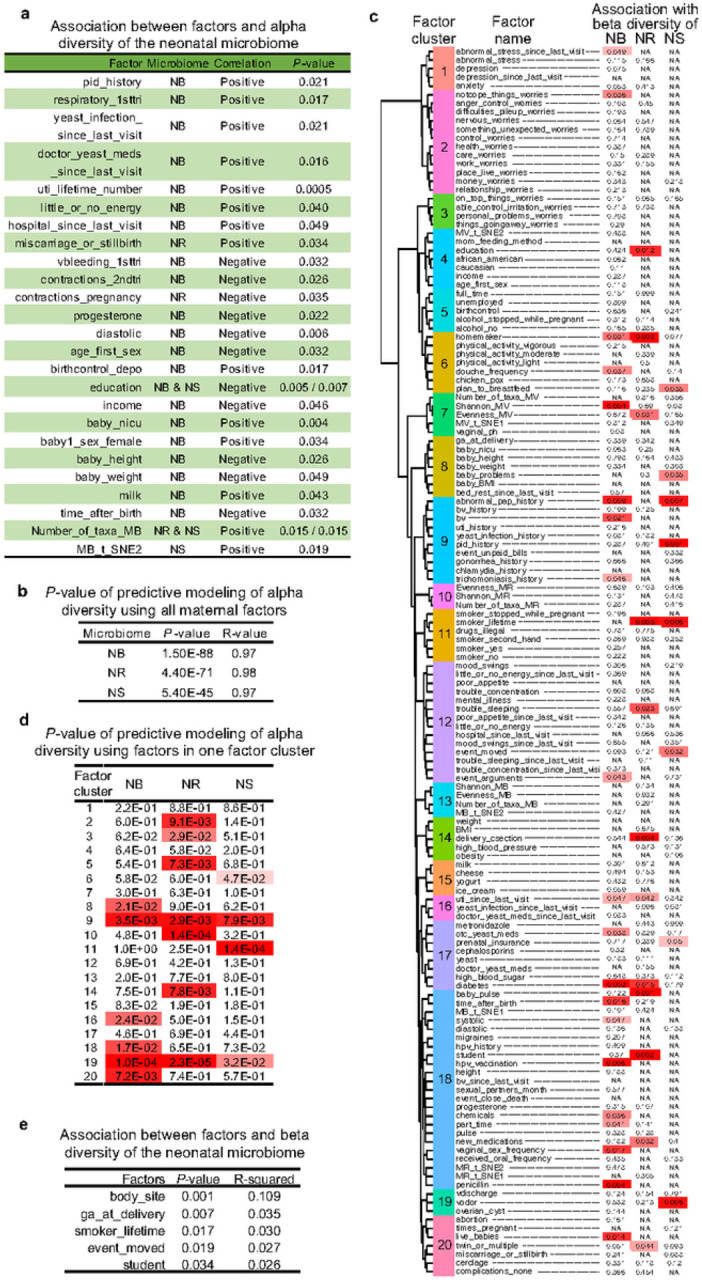
Factors associated with the neonatal microbiomes. **(a)** The association of alpha diversity of the NB, NR, and NS microbiomes with factors in the metadata calculated by the Mann-Whitney U test or linear regression (see Methods). The correlation was determined by the linear regression slope or by comparing the median values of alpha diversity in matched participants with or without a host characteristic (see Fig. S7). Details of factor annotations, case numbers, and the significance of the associations are provided in Supplementary Data 2. (**b)** Predictive models were built with the alpha diversity of the neonatal microbiomes as a dependent variable and all maternal factors in the metadata as independent variables using the random forest algorithm. The prediction accuracy tested by linear regression between the predicted and true values of the Shanon indexes and evaluated by a *P*-value representing significance and an R-value evaluating correlation coefficient is shown (see Fig. S8). (**c)** The clustering of the maternal and neonatal factors based on Spearman’s correlation among these factors is exhibited on the left panel (see Fig. S9 and Supplementary Data 5). The *P*-values of the association between the beta diversity of the neonatal microbiomes and factors in the metadata calculated by the Adonis multivariate testing are shown on the right panel (see Supplementary Data 5). (**d)** *P*-values of predictive models built with the alpha diversity of the neonatal microbiomes as a dependent variable and one cluster of factors shown on the left panel of Fig. 3c as independent variables using the random forest algorithm. The experimental designs and sample sizes for the results shown in Fig. 3a–d are illustrated in Fig. S1 b and Supplementary Data 1. (**e)** The association between beta diversity of the neonatal microbiomes and factors in the metadata calculated by the Adonis multivariate test with one more factor ‘body site’ involved as an independent variable. The *P-* and R-squared values illustrate the significance and importance of the factors in the test. The experimental design is shown in Supplementary Data 1 sheet 7, which contains 20 sets of metadata and paired NB, NR, and NS microbiomes.

The association between the alpha diversity of each neonatal microbiome and each factor in the metadata was measured by the Mann-Whitney U test or linear regression (see Methods). These maternal factors were mainly associated with the alpha diversity in the NB microbiome, i.e., yeast infection, histories of pelvic inflammatory disease and urinary tract infections, and other maternal diseases and disease histories; issues during pregnancy including contractions, vaginal bleeding, and progesterone administration (as previously reported^29^); behaviors including the age of the first sexual intercourse^30^ and birth control by Depo-Provera injections^31^; environmental factors involving education and annual household income; neonatal factors, e.g., admission to the NICU, baby’s sex, height and weight (Fig. 2a and S7). All the factors related to adverse maternal and neonatal health were associated with higher alpha diversity, whereas those associated with pregnancy and better environmental conditions were associated with a lower alpha diversity in the NB microbiome. These results suggested that a complex NB microbiome was associated with the mothers’ and neonates’ sub-optimal health state. Interestingly, in contrast to males, female neonates exhibited a higher alpha diversity in the NB microbiome. One possible explanation is that potential differences in the neonatal immune systems associated with sex could diversify the NB microbiomes^32–34^. The statistical powers of sample sizes in most of these associations are above 0.9 (Supplementary Data 2).

Alternatively, a non-linear regression analysis using a Leave-One-Out Cross-Validation strategy and the random forest algorithm was performed to predict the alpha diversity of the neonatal microbiomes with multiple variables, and the performance of the prediction was evaluated by linear regression between predicted and true values of the alpha diversity. When all the maternal factors in the metadata were applied as independent variables, the alpha diversities of the NB, NR, and NS microbiomes were accurately predicted, which implied the causal relationship between maternal health and the complexity of the neonatal microbiome (Fig. 2b and S8). Additionally, the factors in the metadata were grouped into twenty clusters based on the Spearman’s correlation among the factors (left panel in Fig. 2c, Fig. S9, and Supplementary Data 5), and twenty individual models were generated using factors in one cluster as independent variables in each model. The predicted values in the models built with factor clusters 9 and 19 are significantly linearly correlated with the true alpha diversity values of the NB, NR, and NS microbiomes (Fig. 2d), suggesting that some of the factors included in microbial infections and infection histories as well as abnormal vaginal odor and discharge on the last visit of pregnancy and presence of ovarian cysts are universal factors that impact the alpha diversity of all three neonatal microbiomes.

The association between the beta diversity of each of the neonatal microbiomes and the factors in the metadata was quantified by the Adonis test containing all the factors as independent variables (Supplementary Data 5, see details in Methods). Several factors influenced the beta diversity of one of the NB, NR, and NS microbiomes as follows: several maternal mental stresses; employment status, e.g., homemaker or student; environmental factors, including education; behaviors, e.g., vaginal douching, smoking, and moving to a new address; diseases and disease histories; e.g., abnormal Pap smear, bacterial vaginosis, and diabetes; C-section; the complexity of the MV microbiome; neonate factors involving baby’s pulse and health problems postpartum; and others (Fig. 2c). The time after birth was only significantly associated with the NB microbiome, implying a faster change of the NB microbiome than the NR and NS microbiomes. The impact of C-section^3^·^11^·^13–17^, diabetes^17,24^, smoking^36^, mental health, and antibiotics^24^ on early-life microbiomes have been reported previously.

Another approach to quantify the influence of body site on the beta diversity of the neonatal microbiomes used twenty sets of paired NB, NR, NS microbiomes, metadata (Supplementary Data 1 sheet 7), and an additional ‘body site’ factor that indicated the niche of the neonatal microbiomes. According to the highest R-squared value, body site was the most important factor in the Adonis analysis, suggesting the importance of micro-environments on the maturation of the neonatal microbiomes (Fig. 2e).

Similar to a previous study^9^, more similar taxa were found in the paired mother-neonate dyads than in the unpaired dyads, but none of the observations were statistically significant (Fig. S10 and Supplementary Data 1 sheet 6), implying that a vertical mother-neonate microbe transmission had limited influence on the neonatal microbiomes within three days postpartum.

### Mediation Of Maternal Lipids On The Association Between Maternal Factors And The Neonatal Microbiome

**Figure 3:**
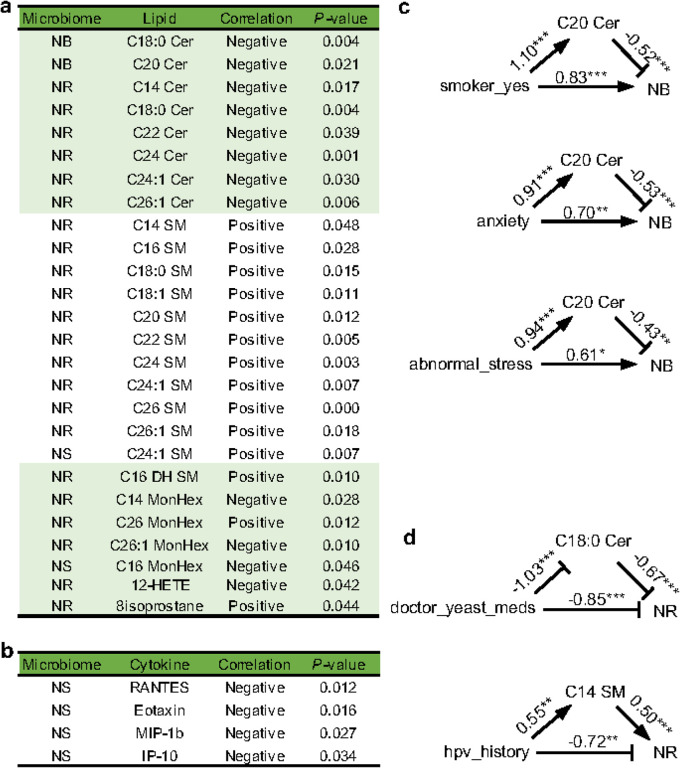
Mediation of maternal lipids on the association between maternal factors and the neonatal microbiome. The association of the alpha diversity of the neonatal microbiomes with lipids **(a)** and cytokines **(b)** in the maternal vaginal fluid evaluated by Pearson’s correlation test is shown. The correlation was determined by the R-values of Pearson’s correlations. Maternal lipids mediating the impact of maternal factors on the NB **(c)** and NR **(d)** microbiomes are illustrated. The mediation effect was tested by structural equation modeling. The degree and significance of the correlations are shown. * *P*-value ≤ 0.05, ** *P*-value ≤ 0.01, and *** *P*-value ≤ 0.001 (see details, i.e., abbreviations, case numbers, the significance of the correlations, and the explanation of the mediation test, in Supplementary Data 6).

Pearson’s correlation analysis showed that twenty-six lipids and four cytokines in the maternal vaginal fluid were associated with the alpha diversity of the neonatal microbiome, particularly the gastrointestinal microbiome (Fig. 3a and b and Supplementary Data 6). Interestingly, the alpha diversity of the NR microbiome was negatively correlated with six ceramides but positively correlated with ten sphingomyelins. Higher concentrations of sphingomyelins and lower levels of ceramides have been reported to be associated with decreased adaptive immune responses^37^. Thus, these observations are consistent with the hypothesis that a more complex NR microbiome is associated with an optimal maternal health condition and a less active adaptive immune system. Similarly, a more complex NS microbiome was correlated with lower concentrations of four cytokines that were associated with maternal inflammation (Fig. 3b). The statistical powers of sample sizes in all the significant associations are higher than 0.99 (Supplementary Data 6).

Our mediation analysis suggested that smoking, anxiety, and abnormal stress of the mothers increased the complexity of the NB microbiome, but meanwhile promoted the concentration of C20 ceramide and, as a result, attenuated the increase of the complexity of the NB microbiome (Fig. 3c and see details in Supplementary Data 6). The uptake of yeast infection medication and the HPV history of the mothers lowered the complexity of the NR microbiome. However, the influence of yeast infection medication and HPV history on the NR microbiome was attenuated by modulating the concentration of C18:0 ceramide and C14 sphingomyelin, respectively (Fig. 3d and see details in Supplementary Data 6). Since a complex NB microbiome has been associated with sub-optimal states of the mothers and neonates (Fig. 2) and high complexity of the gut microbiome is considered a hallmark of gut health^48^, the modulation of the maternal lipids is a protective mechanism by which the mothers limit the adverse impact of smoking, anxiety, abnormal stress, yeast infection medication, and HPV history on the neonatal microbiomes.

### Maternal And Neonatal Factors Associated With The Risk For Nicu Admission

**Figure 4:**
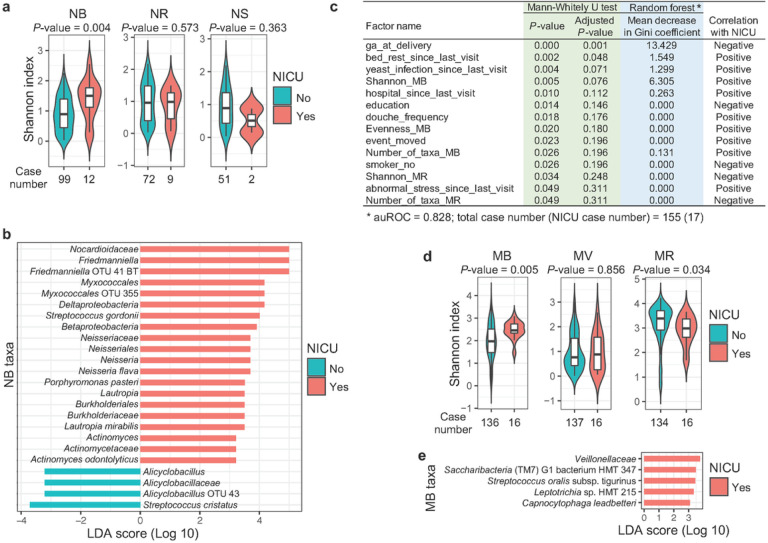
Maternal and neonatal microbiomes and maternal factors associated with NICU admission. The maternal and neonatal microbiomes and metadata were collected on the last visit of pregnancy and the first visit after childbirth, respectively, with matched NICU information in the metadata. (**a)** The association between the alpha diversity of the neonatal microbiomes and the NICU admission quantified by the Mann-Whitney U test is shown. (**b)** The differential abundance of the NB microbiome associated with the NICU admission was tested by the LEfSe analysis. The differences with LDA scores no less than three and *P*-values no larger than 0.001 are shown (see Supplementary Data 7). (**c)** The association between the maternal factors and the NICU admission measured by the Mann-Whitney U test and a random forest algorithm is illustrated (see Methods). The correlation was determined by comparing the median value of a maternal characteristic matched with neonates admitted to the NICU with that not admitted to the NICU or by comparing the NICU admission rate of neonates in matched mothers with or without a characteristic (see Supplementary Data 7). (**d)** The association between the alpha diversity of the maternal microbiomes and the NICU admission quantified by the Mann-Whitney U test is shown. (**e)** The differential abundance of the MB microbiome associated with the NICU admission tested by the LEfSe analysis.

NICU admission rates have risen from 6.62% in 2008 to 9.07% in 2018 in the United States^39^ associated with increased incidence of very low birthweight neonates^40^. Not surprisingly, the NICU admission of the neonates was associated with lower birthweight, height, and BMI (Fig. 2c, S9, and Supplementary Data 5). The alpha diversity of the NB microbiome but not that of the NS or NR microbiome was significantly higher in the neonates being admitted to the NICU (Fig. 4a) and also in the paired mothers with microbial infectious diseases (Fig. 2a). Differential abundance analysis using the LEfSe^41^ showed that several taxa, including some potential pathogens; e.g., *Neisseria* spp., and *Actinomyces*spp.^42^, and several other microbes were enriched in the oral cavity of the neonates admitted to the NICU, but *Streptococcus cristatus,* a commensal oral microbe that inhibits the colonization of the oral pathogen *Porphyromonas gingivalis*^43^, was enriched in the controls (Fig. 4b and Supplementary Data 7). Hence, these results argue that a sub-optimal NB microbiome with higher complexity is associated with NICU admission.

The Mann-Whitney U test illustrated that several maternal factors, e.g., earlier gestational age at delivery, abnormal bed rest, yeast infection, hospitalization, higher frequency of vaginal douching, change of residence, smoking, abnormal stresses, and lower levels of education, were associated with an increased risk of NICU admission (Fig. 4c and Supplementary Data 7). This association was also examined by establishing a machine-learning model using the random forest algorithm and a cross-validation strategy as previously described^44^. The importance of variables in the model quantified by the mean decrease in Gini coefficient showed that gestational age at delivery had the strongest association with NICU admission, an unsurprising finding reflecting that most babies born prematurely are admitted to the NICU^45^. Both the Mann-Whitney U test and the machine learning method indicated that a sub-optimal maternal health condition, e.g., yeast infection and hospitalization, associated with higher alpha diversity of the NB (Fig. 2a), were also risk factors for NICU admission (Fig. 4c).

Interestingly, mothers whose babies would be admitted to the NICU had more complex oral microbiomes and less complex rectal microbiomes before childbirth (Fig. 4d). A higher alpha diversity of the oral microbiome has been associated with two most prevalent oral diseases, i.e., periodontitis and dental caries^34,46^, but a higher complexity of the gut microbiome is generally considered a hallmark of gut health^38^. Additionally, the LEfSe analysis showed that *Veillonellaceae* and a *Saccharibacteria* (TM7) sp. were enriched in the MB microbiome with matched neonates who would be admitted to the NICU (Fig. 4e and Supplementary Data 7) and several *Veillonellaceae* and TM7 spp. have been associated with periodontitis^46^ and preterm birth^26^. Thus, these data illustrated that sub-optimal MB and MR microbiomes were risk factors for NICU admission.

## Discussion

Our data indicate that by 24–48 hours after birth, the NR and NS microbiomes tend to exhibit more similarity to each other than to any maternal microbiome (Fig. 1), suggesting that a vertical mother-neonate microbe transmission is not the most important factor modulating the development of the NR and NS microbiomes after childbirth. The NB microbiome at 24 hours is more similar to the MB microbiome, but the association between the NB and paired MB microbiomes was not detected (Fig.S3e). Additionally, the Adonis test showed that body niche was most important in modulating the neonatal microbiome (Fig. 2e). Thus, the high similarity between the NB and MB microbiomes on days 1 and 2 (Fig. 1 b) is probably due to similar micro-environments in the oral cavity of the neonates and mothers rather than microbe transmission from the MB to the NB microbiome.

Since the oral cavity is the gateway of the human body, local micro-environments in the oral cavity might be more easily affected by the external environment than that in the gut, which could result in our observation of a higher similarity between the NB-MB microbiomes than the NR-MR microbiomes. (Fig. 1b and S3). Microbes could be more easily orally seeded during breastfeeding or by oral contact with other fomites. Since anaerobes are abundant in newborns within one day postpartum^9^, a higher level of oxygen in the oral cavity compared with that in the gut could also lead to a faster change of the NB microbiome. However, the lack of similarity between the MR and NR/NS microbiomes also reflects that the neonatal gastrointestinal tract has a physiology different from that of mature women.

The early-life neonatal microbiomes are influenced by multiple maternal and neonatal factors (Fig. 2). In our study, many of the maternal factors were uniquely associated with only one neonatal microbiome, e.g., the association between C-section and beta diversity of the NR microbiome (Fig. 2c). We assume this is due to the differences in micro-environments in the neonate. Alternatively, it could be due to the relatively small numbers in our cohort or other unknown reasons. However, it seems that microbial infections in the female reproductive tract are universal factors that modulate all the studied neonatal microbiomes as well as the risk of NICU admission (Figs. 2 and 4c), probably because diseases in the female reproductive tract could easily influence the environment of the fetus.

It is unclear how maternal factors, e.g., diseases, stress, and mood^24^, impact the neonatal microbiomes. Our results exhibit direct evidence that immunity-related lipids mediate the association between maternal factors and the neonatal microbiome (Fig. 3). Taken together with previous studies showing the association between maternal immune-related metabolites and the development of the fetal and neonatal immune system^16,47^ and the interaction between the neonatal immune system and the neonatal microbiome^48^, the immunity-related maternal lipids may influence the neonatal microbiome through modulating the neonatal immune system.

Previous studies have shown that bacteria can spread hematogenously from the oral cavity to the uterus, and periodontal disease has been associated with preterm birth^49^. Thus, a MB microbiome with higher complexity and potential pathogens, e.g., TM7, could increase the risk of preterm birth and NICU admission (Fig. 4d and e). Maternal factors, e.g., microbial infections, have been reported to influence the gestational age at delivery or the risk for preterm birth^35^ and could subsequently impact the health state, e.g., the NICU admission, birthweight (Fig. 3c and 4c), and the development of the neonatal immune system^50^.

## Methods

### Cohort

Data used in this study were produced under the umbrella of the Multi-Omic Microbiome Study-Pregnancy Initiative (MOMS-PI) project^26,27^, which enrolled ~ 1500 pregnant women with the goal of studying the contribution of the vaginal microbiome to adverse outcomes of pregnancy, including preterm birth. Here, we focused on the maturation of early-life neonatal microbiomes and variables influencing this process. The studied dataset was collected from 164 mother-neonate dyads, including 16S rRNA taxonomic profiles from neonatal buccal, rectal, and stool sites, maternal buccal, rectal, and vaginal niches in the pregnant women, maternal and neonatal clinical and other metadata, and lipid and cytokine expression levels in the vaginal fluid of the pregnant women (Supplementary Data 1 and 2). Maternal samples and metadata were from the last pregnancy visit (Supplementary Data 1). Neonatal metadata was collected on the first visit after childbirth, and neonatal samples were collected on the first visit on day 0 (within 24 hours postpartum), day 1 (24 ~ 48 hours postpartum), and day 2 (48 ~ 72 hours postpartum), but there were no neonatal stool samples from day 0. Metadata of the 164 mother-neonate dyads include the time of sample collection during or after pregnancy, gestational age at delivery, delivery method, maternal disease records, adverse outcomes of pregnancy, maternal stress level, body mass index, birth control methods, drug use, racioethnicity, diet, economic status, etc. (Supplementary Data 2). The experimental design and case number for each analysis are shown in Fig. S1 and Supplementary Data 1 and 2.

### Data Processing

Raw 16S rRNA sequencing data were treated by quality control, trimming, merging paired sequence reads, and removing human reads as previously described^26,51^. For better taxonomic profiling of the 16S rRNA sequencing data to species level, a new 16S rRNA V1-V3 region database was created based on the Greengenes database version gg_13_5 (https://greengenes.secondgenome.com/)^52^ and the HOMD database version 15.1 (https://www.homd.org/)^53^. The 16S rRNA sequences in the Greengenes and HOMD databases were mixed and sorted in the following order. The sequences with taxonomic annotations at the species level in the Greengenes database had the highest priority, followed by all the sequences in the HOMD database, the Greengenes sequences with annotations at the genus level, and then the Greengenes sequences with annotations at levels higher than genus level. The V1-V3 region of the full-length 16S rRNA sequences was extracted using the V-Xtractor^54^. Finally, V1-V3 sequences in the database with a similarity higher than 97% are filtered using USEARCH^55^ so that only one remains in the database. The trimmed, merged, and filtered 16S rRNA raw data were assigned to the species level using the new 16S rRNA V1-V3 region database (https://github.com/GregoryBucklab/Neonatal_microbiome_project/16S_rRNA_V1-V3_database_VCU_10_2022) to generate the feature tables of the microbiomes. Samples with less than 5,000 total reads in the feature tables were eliminated from this study. Pretreated 16S rRNA sequencing data were aligned to the new database for taxonomic assignment. An alignment with a similarity lower than 97% was assigned as ‘BT’ (below the threshold).

### Diversity Analysis

The experimental design and case numbers are outlined in Fig. S1 a and Supplementary Data 1. Samples of the maternal microbiomes that were included herein were collected on the last prenatal visit, and neonatal samples were collected on the first visits of days 0 (0–24 hours), 1 (24–48 hours), and 2 (48–72 hours). Before diversity analysis, the feature tables were pre-filtered by a taxon threshold in which only taxa with relative abundances higher than 0.1% in more than 5% of samples or no less than 1 read in more than 15% of samples were kept. The feature tables were normalized by rarefaction to the depth of the lowest number of reads in the samples (5,000) for diversity analysis. Alpha diversity, quantified by the Shannon index, evenness, and the number of observed taxa, were evaluated using the ‘vegan’ package in R^56^. The two-sided Mann-Whitney U test was used to test the difference between alpha diversities of two microbiomes. Beta diversity was measured and visualized by a t-distributed stochastic neighbor embedding (t-SNE) of Bray-Curtis distances using the ‘Rtsne’ package in R^57^. Alternatively, Bray-Curtis distances between each microbiome dyad were tested using the ‘vegan’ package in R^56^, and the Euclidean distances among median values of the Bray-Curtis distances were clustered and visualized by the ‘pheatmap’ package in R with the ‘complete’ clustering method. The difference in beta diversity between two microbiomes quantified by the Bray-Curtis distance was measured by a PERMANOVA analysis using the ‘adonis2’ function in the ‘vegan’ package^56^. The change in beta diversity with the time after birth was quantified by the two-sided Kruskal-Wallis test. The difference between within-group Bray-Curtis distances of two microbiomes was measured by the multiple response permutation procedure (MRPP) test using the ‘mrpp’ function in the ‘vegan’ package in R. Lines in all the boxplots in this study represent maximum, 75% quantile, median, 25 quantile, and minimum values from top to bottom.

### Change Of Relative Taxon Abundance In The Neonatal Microbiome With The Time After Birth

The design of this analysis is the same as that in ‘Diversity analysis’ in the Methods, but only the neonatal microbiomes were involved. The same taxon threshold was applied to pretreat the 16S rRNA feature tables introduced in the diversity analysis. Differential abundance analysis was performed using the ‘ALDEx2’ package in R^28^. The adjusted P-value of relative abundance differences was tested by the ‘aldex.ttest’ function^28^ using the two-sided Mann-Whitney U test value, followed by the Benjamini-Hochberg correction. The relative abundance change was measured by the ‘aldex.effect’ function and quantified by the per-feature median difference between the two conditions.

### Correlation Networks Of Taxa Within Each Neonatal Microbiome

The design of this analysis is the same as that in ‘Diversity analysis’ in the Methods, but only the neonatal microbiomes were involved. The same taxon threshold was applied to pretreat the 16S rRNA feature tables introduced in the diversity analysis. The treated features tables were normalized by the Centered Log-Ratio transformation. The Spearman’s correlation between each taxa dyad in the neonatal microbiomes within three days postpartum was evaluated by the ‘rcorr’ function in the ‘Hmisc’ package in R, which generated a *P*-value and an R-value for the significance and strength of the correlation, respectively. The *P*-values were adjusted by the Benjamini-Hochberg correction. The R-values with adjusted *P*-values higher than 0.05 were adjusted to zeros, which was an attempt to remove insignificant correlations in the clustering analysis. Correlations among taxa in each microbiome profile were color-coded by the adjusted R-values. The Euclidean distance and the ‘complete’ clustering method were applied for the clustering of the adjusted R-values using the ‘pheatmap’ package in R. Taxa were classified into three groups according to their clustering in the networks using the ‘cutree’ function in R.

### Missing Samples In The Longitudinal Cohort

Because missing samples exist in the longitudinal cohort (Fig. S1a and Supplementary Data 1 sheet 2), sample numbers from different participants are not the same, which could lead to a bias in sample collection in the diversity and composition analyses. Thus, the Adonis test was performed to explore the impact of participants on the beta diversity of the neonatal microbiomes. The *P*-values for the influence of participants on the NB, NR, and NS microbiomes within three days postpartum were 0.751, 0.476, and 0.001, respectively, suggesting that the bias in sample collection had a potential impact on the analysis of the beta diversity and composition of the NS microbiome.

### Association Between Alpha Diversity Of The Neonatal Microbiome And Maternal And Neonatal Factors

Maternal metadata and microbiomes on the last visit of pregnancy and paired neonatal metadata, and microbiomes on the first visit after birth were selected in this analysis (Fig. S1b and Supplementary Data 1 sheet 2–5). There are generally three forms of values, i.e., ordinal values with two levels, ordinal values with more than two levels, and numerical values, in the metadata (Supplementary Data 2). The ordinal values with two levels contain values ‘No’ and ‘Yes’. The ordinal values with more than two levels were converted to numerical values (see details in Supplementary Data 2 sheet 1 column E). For example, the numbers 0 ~ 5 in the subject ‘douche_frequency’ represent ‘never’, ‘less than once a month’, ‘1–3 times per month’, ‘2–6 times per week’, ‘once a week’, and ‘once a day’, respectively. Alpha diversity was quantified by the Shannon index in this analysis. The association between alpha diversity of the neonatal microbiome and factors in the metadata in the form of ordinal values with two levels was measured by a two-sided Mann-Whitney U test. The association between alpha diversity of the neonatal microbiome and factors with numerical values was measured by linear regression using the ‘lm’ function in R. The correlation between metabolites in the vaginal fluid and alpha diversity of the neonatal microbiome was calculated by linear regression using the ‘lm’ function in R.

### Clustering Of Factors In The Metadata

Maternal metadata and microbiomes on the last visit of pregnancy and paired neonatal metadata, and microbiomes on the first visit after birth were selected in this analysis (Fig. S1b and Supplementary Data 1 sheet 2–5). The ordinal values with two levels in the metadata were converted to numeric values 0 and 1. The factors with more than 25% missing values were excluded from the following analyses, leaving 142 factors, as shown in Fig. 2c and Supplementary Data 5. The Spearman’s correlation among factors in the metadata was measured, which generated a *P*-value and an R-value for the significance and strength of the correlation among the metadata, respectively. The *P*-values were adjusted by the Benjamini-Hochberg correction. The R-values with adjusted *P*-values higher than 0.05 were adjusted to zeros to remove insignificant correlations in the clustering analysis. According to absolute values of the adjusted R-values in Spearman’s correlation analysis, the factors were clustered by testing the Canberra distances and applying the ‘ward.D’ clustering method using the ‘pheatmap’ package in R and further divided into 20 groups using the ‘cutree’ function in R.

### Predictive Modeling Of Alpha Diversity Of The Neonatal Microbiome Using Factors In The Metadata

Maternal metadata and microbiomes on the last visit of pregnancy and paired neonatal metadata, and microbiomes on the first visit after birth were selected in this analysis (Fig. S1b and Supplementary Data 1 sheet 2–5). The ordinal values with two levels in the metadata were converted to numeric values 0 and 1. The factors with more than 25% missing values were excluded from the following analyses, leaving 142 factors, as shown in Fig. 2c and Supplementary Data 5. Missing values in the 142 factors were imputed using the ‘mice’ package in R^58^. Alpha diversity was quantified by the Shannon index. The alpha diversity of the neonatal microbiomes was predicted by a Leave-One-Out Cross-Validation strategy and the random forest algorithm using the ‘caret’ package in R^59^ with the ‘method’ and ‘importance’ parameters setting as ‘ranger’ and ‘permutation’, respectively. The prediction accuracy was evaluated by linear regression between the predicted and true values of the Shannon indexes. The *P*-value representing significance and R-value evaluating correlation coefficient in the linear regression was measured by the ‘lm’ function in R.

### Association Between Beta Diversity Of The Neonatal Microbiome And Factors In The Metadata

Maternal metadata and microbiomes on the last visit of pregnancy and paired neonatal metadata, and microbiomes on the first visit after birth were selected in this analysis (Fig. S1b and Supplementary Data 1 sheet 2–5). The ordinal values with two levels in the metadata were converted to numeric values 0 and 1. The factors with more than 25% missing values were excluded from the following analyses, leaving 142 factors, as shown in Fig. 2c and Supplementary Data 5. Missing values in the 142 factors were imputed using the ‘mice’ package in R^58^. The association between each factor in the metadata and beta diversity of one neonatal microbiome was quantified by the ‘adonis2’ function in the ‘vegan’ package^56^ with the following formula: 16S rRNA profiles of one type of the neonatal microbiomes ~ Variable x. The results of this analysis are shown in Supplementary Data 5 sheet 5 in the columns for tests with single independent variables. The factors in the metadata were sorted by ranking the *P*-values in the tests with single independent variables from low to high, and the ordered factors were input into tests with 142 independent variables using the ‘adonis2’ function in the ‘vegan’ package with a formula (16S rRNA profiles of one neonatal microbiome ~ Factor 1 + Factor 2 + … + Factor 142). The results of the tests with 142 independent variables are shown in Fig. 2c and Supplementary Data 5 sheet 5 in the columns for tests with 142 independent variables. The association between factors in the metadata and beta diversity of all three neonatal microbiomes was tested by adding a factor ‘body site’ representing the niche of the neonatal microbiomes. The design of this analysis contains 20 sets of paired NB, NR, and NS microbiomes and metadata (Supplementary Data 1 sheet 7). The adonis tests were performed as introduced above. The results of tests with 142 independent variables are shown in Fig. 2e and Supplementary Data 5 sheet 6.

### Coexistence Of Taxa In Each Neonatal-maternal Microbiome Dyad

Paired maternal and neonatal microbiome dyads with maternal samples collected on the last visit of pregnancy and neonatal samples collected on the first visit after birth were selected, and sample pair lists are shown in Supplementary Data 1 sheet 6. Additionally, only data from vaginally delivered neonates and paired mothers were included in this analysis. The same taxon threshold was used to pretreat 16S rRNA feature tables as introduced in the diversity analysis. The presence of a taxon in a sample was defined as a number of reads larger than 0. The difference between the numbers of coexisted taxa in paired and unpaired maternal-neonatal sample dyads was evaluated by the two-sided Mann-Whitney U test.

### Mediation Analysis

The mediation effect of metabolites on the association between factors in the metadata and alpha diversity of the neonatal microbiome was evaluated by the ‘Structural Equation Modeling’ (‘sem’) function in the ‘lavaan’ package in R^60^.

### Factors And Microbiomes Associated With The Nicu Admission

The maternal and neonatal microbiomes were collected on the last visit of pregnancy and the first visit after childbirth, respectively, with matched NICU information in the metadata. The method for alpha diversity analysis was the same as that shown in ‘Diversity analysis’ in the Methods. The two-sided Mann-Whitney U test for measuring the association between maternal factors and the risk of NICU admission was the same as that introduced in ‘Association analysis between alpha diversity of the neonatal microbiome and maternal and neonatal factors’ in the Methods. The random forest analysis was performed using the maternal factors as independent variables and the outcome of the NICU admission as a dependent variable. Data on the maternal factors were pretreated as described in ‘Predictive modeling of alpha diversity of the neonatal microbiome using factors in the metadata’ in the Methods. A random forest algorithm and the ‘randomForest’ package in R^60^ were applied to create the predictive model as previously described^44^. The quality of the model was measured by the area under the receiver operating characteristic curve (auROC), and the importance of variables in the model was measured by the mean decrease in Gini coefficient. The differential abundance of microbiomes associated with the NICU admission was measured by the LEfSe^41^ analysis.

### Statistical Power Analysis

In the analysis using the two-sided Mann-Whitney U test, the distribution of the dependent variable was tested by the ‘descdist’ function in the ‘fitdistrplus’ package, and the statistical power was measured by the ‘shiehpow’ function in the ‘wmwpow’ package. In linear regression analysis, the statistical power was measured by the ‘wp.regression’ function in the ‘WebPower’ package. In the analysis of the association between factors in the metadata and beta diversity of the neonatal microbiomes using the adonis test, the statistical power was measured using the ‘micropower’ package. More details are shown in the function ‘adonis_power’ available on GitHub (https://github.com/GregoryBucklab/Neonatal_microbiome_project). The Type I error in all the statistical power analyses was set as 0.05.

## Data Availability

Raw 16S rRNA sequences, cytokine, lipidomics data, and limited metadata of the Multi-Omic Microbiome Study-Pregnancy Initiative (MOMS-PI) project26,27 are available from the HMP DACC (https://portal.hmpdacc.org). Controlled-access data for all subjects in the MOMS-PI project are available at National Center for Biotechnology Information’s controlled-access dbGaP (study no. 20280; accession ID phs001523. v1 .p1) and the SRA under BioProject IDs PRJNA326441, PRJNA326442, and PRJNA326441. Further information and requests for resources should be directed to and will be fulfilled by the lead contact, Gregory A. Buck (gregory.buck@vcuhealth.org). All the codes are available on GitHub (https://github.com/GregoryBucklab/Neonatal_microbiome_project). All the tools used in this study are shown in Supplementary Table 1.
